# Knowledge translation to fitness trainers: A systematic review

**DOI:** 10.1186/1748-5908-5-28

**Published:** 2010-04-15

**Authors:** Dawn Stacey, Michael Hopkins, Kristi B Adamo, Risa Shorr, Denis Prud'homme

**Affiliations:** 1 School of Nursing, Faculty of Health Sciences, University of Ottawa, Ottawa, ON, Canada; 2 Clinical Epidemiology Program, Ottawa Hospital Research Institute, Ottawa, ON, Canada; 3 School of Human Kinetics, Faculty of Health Sciences, University of Ottawa, Ottawa, ON, Canada; 4 Healthy Active Living and Obesity Research Group, Children's Hospital of Eastern Ontario Research Institute, Ottawa, ON, Canada; 5 Library Services, The Ottawa Hospital, Ottawa, ON, Canada

## Abstract

**Background:**

This study investigates approaches for translating evidence-based knowledge for use by fitness trainers. Specific questions were: Where do fitness trainers get their evidence-based information? What types of interventions are effective for translating evidence-based knowledge for use by fitness trainers? What are the barriers and facilitators to the use of evidence-based information by fitness trainers in their practice?

**Methods:**

We describe a systematic review of studies about knowledge translation interventions targeting fitness trainers. Fitness trainers were defined as individuals who provide exercise program design and supervision services to the public. Nurses, physicians, physiotherapists, school teachers, athletic trainers, and sport team strength coaches were excluded.

**Results:**

Of 634 citations, two studies were eligible for inclusion: a survey of 325 registered health fitness professionals (66% response rate) and a qualitative study of 10 fitness instructors. Both studies identified that fitness trainers obtain information from textbooks, networking with colleagues, scientific journals, seminars, and mass media. Fitness trainers holding higher levels of education are reported to use evidence-based information sources such as scientific journals compared to those with lower education levels, who were reported to use mass media sources. The studies identified did not evaluate interventions to translate evidence-based knowledge for fitness trainers and did not explore factors influencing uptake of evidence in their practice.

**Conclusion:**

Little is known about how fitness trainers obtain and incorporate new evidence-based knowledge into their practice. Further exploration and specific research is needed to better understand how emerging health-fitness evidence can be translated to maximize its use by fitness trainers providing services to the general public.

## Background

Lack of physical activity (or sedentarity) is associated with an increased risk of health problems and chronic diseases such as obesity, type 2 diabetes, cardiovascular disease, cancer, osteoporosis, and depression [1]. Adoption of regular physical activity is strongly recommended for the prevention and treatment of obesity and associated co-morbidities, with national guidelines recommending a combination of endurance, strength, and flexibility training [1-3].

Fitness trainers are a resource for the general public to obtain exercise information, exercise prescription, and guidance. Fitness trainers, often called personal trainers, specialize in the assessment of an individual's fitness level and the design and supervision of exercise programs tailored to individual fitness goals such as weight reduction [4]. Credentialing of fitness trainers is offered through many fitness organizations, and their requirements range from brief online courses to university degrees and/or stringent certifying examinations [5-7]. In 2006, there were approximately 235,000 fitness workers, including fitness trainers, registered in the United States and these numbers are expected to increase by 27% between 2006 and 2016 [4]. However, accessibility to fitness trainers depends on an individual's financial ability to pay for access to the facility and/or the trainer for advice.

The integration of fitness trainers into primary care has been studied. A review of eighteen studies examining physician-to-trainer 'exercise referral schemes' found that they have a small effect on increasing physical activity level in patients [8]. Another review of exercise interventions in primary care revealed that interventions performed by allied health professionals, including exercise specialists, may be more effective than those delivered by a physician alone, potentially due to the enhanced ability of health professionals to provide a more intensive intervention than time-constrained physicians [9]. Indeed, a survey of 500 physicians revealed that they rate themselves as ineffective at helping patients remain physically active and felt too hampered by time constraints to provide effective exercise counseling [10]. Finally, the 2006 Canadian guidelines for management of obesity recommend that inter-professional teams include exercise specialists [11].

Concurrently, there is an increased focus by national research granting agencies on the uptake and use of research findings [12,13]. This process is commonly called knowledge translation (KT), implementation, dissemination, diffusion, or knowledge transfer [13]. KT is defined as 'a dynamic and iterative process that includes synthesis, dissemination, exchange and ethically-sound application of knowledge to improve health, provide more effective health services and products and strengthen the healthcare system' [14]. However, research is not often translated for use in practice, and studies are underway to determine effective interventions for KT.

The Cochrane Effective Practice and Organization of Care (EPOC) Group reports systematic reviews of studies designed to determine effective means for transferring knowledge to healthcare professionals [15]. KT strategies identified in EPOC reviews that have been shown to improve uptake of evidence-based knowledge include practice reminders (14% change in practice), educational meetings (11 to 20%), local opinion leaders (10%), audit and feedback (5%), printed educational materials (5%), and educational outreach (5%) [16]. Unfortunately, no reviews have been identified that target fitness trainers, and it is unclear how or to what extent fitness trainers integrate research findings in their practice.

In summary, although fitness trainers are publicly available to work with individuals to enhance physical activity, there is wide variability in the requirements for certification, including the knowledgebase and ongoing learning. Concurrently, studies are underway to improve fitness among the public, but effective strategies to transfer knowledge into practice is limited to healthcare providers.

The overall aim of this systematic review was to explore approaches or channels for translating scientific evidence-based knowledge to fitness trainers. Specific research questions were: Where do fitness trainers get their evidence-based information? What types of interventions are effective for translating evidence-based knowledge for use by fitness trainers? What are the barriers and facilitators to the use of evidence-based information by fitness trainers in their practice?

## Methods

### Search Protocol

**Figure 1:**
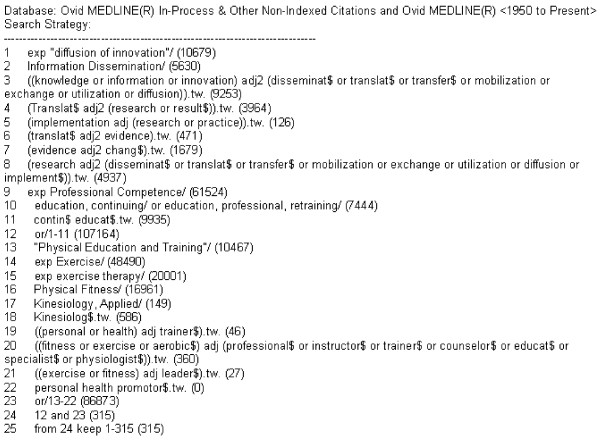
**Medline search strategy**.

The search strategy incorporated key terms related to fitness trainers (*e.g*., personal/fitness/exercise, trainer/instructor/leader/professional) and KT (*e.g*., dissemination, transfer, implementation) [17]. Given the authors' perception that KT to fitness professionals may be limited, a broad search strategy favoring high sensitivity was used (see Figure 1). The following databases were searched to May 2009: Medline, EMBASE, PsycINFO, Sport Discus, CINAHL, Scholars Portal Physical Education interface, and the Cochrane Central Register of Controlled Trials (second quarter, 2009). Additionally, the reference lists of included citations (n = 2) and excluded citations directly pertaining to fitness trainers (n = 9) were examined to search for other relevant publications. These eleven studies were also entered into Pubmed to search for related articles and to crosscheck publications by all study authors. Results were combined and duplicates removed.

### Selection process

The criteria for determining eligibility are described in Table 1. Two authors (DS, MH) independently screened titles and abstracts of all identified citations and, for relevant citations, assessed their eligibility for inclusion. Two authors (DS, MH) independently extracted data of included studies using a standardized form. Quality appraisals, including risk of bias, were based on the Critical Appraisal and Skills Program criteria for qualitative, descriptive, and observational studies [18]. Inconsistencies between reviewers were resolved by consensus at screening, eligibility assessment, and data abstraction.

**Table 1:** Criteria for study inclusion

Criteria:	Included:	Excluded:
Population:	Fitness Trainer working with public. (*i.e*., personal trainer, fitness professional, exercise specialist, fitness leader, health fitness specialist)	• Strength coach • Recreational therapist • Athletic trainer • Nurse, physician, physiotherapist • General public • Other

Focus/Intervention:	KT defined as fitness trainers identifying and using research findings in practice	

Outcomes:	• Improved knowledge • Attitude toward use of evidence • Preferred knowledge sources • Intention/actual use of evidence • Barriers and facilitators to using evidence • Fitness trainer satisfaction with KT intervention	• Current level of knowledge

Study Design:	All	

Language:	English or French	Other languages

### Synthesis process

Data from standardized forms were entered into an Excel database. Data synthesis was guided by the three research questions. For quantitative studies, we reported the results for outcomes as described in the paper aligned to one or more of the research questions. A similar approach was used to report qualitative data as described by the authors. Given the paucity of studies identified, we did not conduct a meta-analysis for similar quantitative outcomes or meta-qualitative analysis to identify themes across studies.

## Results

**Figure 2:**
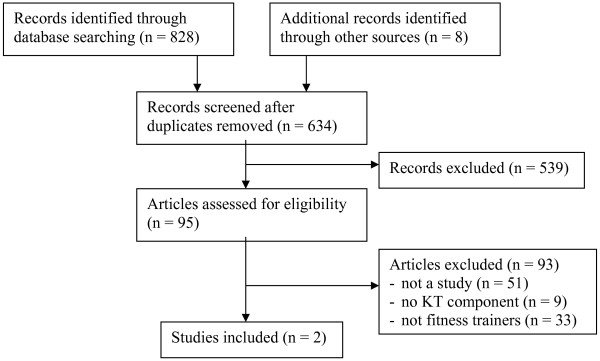
**Flow diagram for screening process**.

Of 626 unique citations identified in the search strategy, and an additional eight studies retrieved through alternative methods, two studies were eligible for inclusion (see Figure 2). One study employed a survey design and the other a qualitative methodology. Of the 93 citations excluded after articles were reviewed, 51 were not studies, 33 were not about fitness trainers (*e.g*., three teachers, six athletic trainers, eight general public, 16 healthcare professionals), and nine did not measure how fitness trainers currently obtain/use evidence-based information (see Table 2).

**Table 2:** Characteristics of excluded studies (n = 42)

First Author, Year	Title	Reason for Exclusion
Dawson, 2001	The ethical beliefs and behaviours of Victorian fitness professionals	No KT to fitness trainers
	
Lobb, 2008	Perceptions of antiobesity medications among personal trainers	
	
Malek, 2002	Importance of health science education for personal fitness trainers	
	
Manley, 2008	Fitness instructors' recognition of eating disorders and attendant ethical/liability issues	
	
Melton, 2008	The current state of personal training: an industry perspective of personal trainers in a small Southeast community	
	
Shields, 2007	Do personal trainers help clients take responsibility for their exercise?	
	
Tulloch, 2006	Physical activity counseling in primary care: who has and who should be counseling?	
	
Wiles, 2008	Exercise on prescription schemes for stroke patients post-discharge from physiotherapy	
	
Wing, 1996	Effects of a personal trainer and financial incentives on exercise adherence in overweight women in a behavioral weight loss program	

Dowda, 2005	Evaluating the sustainability of SPARK physical education: a case study of translating research into practice	Wrong population: children, teachers
	
Kelly, 1998	Preparation and job demographics of adapted physical educators in the United States	
	
Sondag, 1998	Integrating health and physical education: a data-based assessment of theory versus practice	

Graves, 1991	Nutrition training, attitudes, knowledge, recommendations, responsibility, and resource utilization of high school coaches and trainers	Wrong population: coaches, athletic trainers
	
Rockwell, 2001	Nutrition knowledge, opinions, and practices of coaches and athletic trainers at a Division I University	
	
Walker, 2008	Evaluation of athletic training students' clinical proficiencies	
	
Weidner, 2005	Importance and applicability of approved clinical instructor standards and criteria to certified athletic trainers in different clinical education settings	
	
Whitson, 2006	Certified athletic trainers' knowledge and perception of professional preparation involving eating disorders among athletes	
	
William, 2007	Perceptions of elite coaches and sports scientists of the research needs for elite coaching practice	

Boltri, 2008	Diabetes prevention in a faith-based setting: results of translational research	Wrong population: general public
	
Dajpratham, 2007	Knowledge and practice of physical exercise among the inhabitants of Bangkok	
	
Estabrooks, 2008	Determining the impact of Walk Kansas: applying a team-building approach to community physical activity promotion	
	
Faulkner, 2007	Get the news on physical activity research: a content analysis of physical activity research in the Canadian print media	
	
Leslie, 1991	The effect of contingency contracting on adherence and knowledge of exercise regimens	
	
Hooker, 2005	The California active aging community grant program: translating science into practice to promote physical activity in older adults	
	
Kelly, 2007	Translating research into practice: using concept mapping to determine locally relevant intervention strategies to increase physical activity	
	
McNamara, 2008	Online weight training	

Abramson, 2000	Personal exercise habits and counseling practices of primary care physicians: a national survey	Wrong populations: healthcare professional
	
Allenspach, 2007	Patient and physician acceptance of a campaign approach to promoting physical activity	
	
Bowman, 2007	Physical activity advisement practices in diabetes education centres across Canada	
	
Brehm, 1999	Training Health Professionals: A Multidisciplinary Team Approach in a University-based Weight-loss Program	
	
Connaughton, 2001	Graduating medical students' exercise prescription competence as perceived by deans and directors of medical education in the United States	
	
Davis, 2008	Interprofessional continuing health education for diabetic patients in an urban underserved community	
	
Douglas, 2006	Primary care staff's views and experiences related to routinely advising patients about physical activity. A survey	
	
Huang, 2004	The Victorian Active Script Programme: promising signs for general practitioners, population health, and the promotion of physical activity	
	
Kopp, 2008	Proper Exercise and Nutrition kit: use of obesity screening and assessment tools with underserved populations	
	
Kremer, 1995	Physical activity programs offered in substance abuse treatment facilities	
	
Laws, 2004	A new evidence-based model for weight management in primary care: the Counterweight Programme	
	
Mann, 2000	Prescribing exercise for cardiac patients: Knowledge, practices, and needs of family physicians and specialists	
	
McQuigg, 2005	Empowering primary care to tackle the obesity epidemic: the Counterweight Programme	
	
Miller, 2000	Health-related physical fitness knowledge of student allied health professions. Evaluation and the Health Professions	
	
Petrella, 2007	Physical activity counseling and prescription among Canadian primary care physicians	
	
Ruby, 1993	The knowledge and practices of registered nurse, certified diabetes educators: teaching elderly clients about exercise	

### Question one: Where do fitness trainers get their evidence-based knowledge?

Hare and colleagues surveyed a group of 325 American College of Sports Medicine registered fitness professionals working across a range of environments to assess their attitudes, perceptions, and beliefs regarding obesity [19]. Of 25 survey items, one item asked respondents to identify what sources of information they use to obtain knowledge on weight control information. Participants identified textbooks (81%), college class notes (80%), scientific journals (79%), workshops/seminars (78%), past experience (51%), colleagues (49%), and mass media (20%). Participants holding a Doctorate degree (14%) were less like to use mass media as a source of information than those holding a Master's degree or less (86%) (p = 0.04). Participants holding a Master's or Doctorate degree (61%) were more likely to use scientific journals as source of information than those with a Bachelor's degree or less (39%) (p = 0.008). Limitations include the potential for response bias with 66% response rate and the absence of *a priori *identification of units of analysis. Strengths were having an explicit description of the survey development and validation process, appropriate recruitment of participants, and clear reporting of results.

Forsyth and colleagues conducted a qualitative study to examine the knowledge, approaches, and attitudes of fitness instructors dealing with clients seeking weight loss advice [20]. A purposive sample of 10 fitness instructors in New Zealand, with qualifications ranging from none to Master's level preparation, was interviewed. A wide range of knowledge and competency concerning weight control and exercise prescription was found among the participants. All rated keeping current with knowledge as important. Preferred knowledge resources included word of mouth (networking with peers), followed by internet, fitness magazines, seminars, and research papers. Less educated participants were more likely to use the internet and reported difficulty determining accuracy and credibility of information. Limitations included insufficient description of the data analysis process and lack of author reflection regarding personal bias and relationship with participants. Despite limitations, this study provides a clear statement of research aims, used an appropriate study methodology to accomplish the objectives, and provided a rich description of results.

### Question two: What types of interventions are effective for translating evidence-based knowledge to fitness trainers?

No studies identified addressed this question.

### Question three: What are the barriers and facilitators to the use of evidence-based knowledge by fitness trainers in their practice?

No studies identified specifically addressed this question. However, as noted above, education level was identified as a factor influencing knowledge sources and could be considered as a barrier or facilitator to finding research-based evidence for use in practice.

## Discussion

This is the first known systematic review of KT strategies used by fitness trainers. The principal finding is the lack of literature related to this important question. In fact, included studies were limited to description of sources of information used by fitness trainers, and none evaluated interventions for KT to fitness trainers. Furthermore, both studies targeted fitness trainers' perspectives on body weight control issues, and KT was not one of the main objectives.

The most common sources of information used by fitness trainers were textbooks, networking with colleagues, scientific journals, seminars, and mass media [19,20]. These sources reflect a range of quality of the information with only one source, scientific journals, likely to include evidence-based information to inform practice [21]. Textbooks and course notes are considered to be of lower quality given the often absence of peer review and the time delays in publishing, rendering some knowledge out of date quickly. As well, textbooks and notes were likely to be further out-dated for the majority of participants, as the 325 surveyed by Hare *et al*. had been employed for a mean of 10.3 years [19]. From a KT perspective, systematic reviews or practice guidelines that synthesize evidence from multiple studies are identified as the 'unit of knowledge' for moving evidence into practice [13]; however, none of the participants in the two studies specifically identified having used either of these sources of evidence-based knowledge.

Both studies also suggested that fitness trainers with higher levels of education (*e.g*., graduate degrees) are more likely to use scholarly sources of evidence compared to those with lower levels of education who are more likely to rely on mass media, including the internet [19,20]. Of concern is that those using the internet were also described as having difficulty discerning the credibility and quality of information sources [20]. The implication of educational level as a factor influencing choice of information sources is difficult to determine, given that there are little data on the educational qualifications of fitness trainers working with the public. Another study examining current knowledge of 115 health fitness professionals working in fitness facilities in Southern California found that three held Master's degrees (3%), 22 held bachelor's degrees in exercise science (19%), nine held other bachelor's degrees (8%), and the majority (70%) held less than a bachelor's degree [22]. In comparison with the study by Hare *et al*. in which 61% of participants held postgraduate university degrees and worked across a range of environments including hospitals, rehabilitation clinics, and universities. Therefore, it is challenging to determine what proportion of these individuals are actively involved in providing individual exercise counseling, thereby making it difficult to extrapolate our findings to the fitness trainer population at large.

Unfortunately, our systematic review did not identify any KT intervention studies evaluating outcomes such as knowledge uptake, intention to use research in practice, use of evidence in practice, or fitness trainer satisfaction with KT interventions. As one example of KT to fitness trainers, the Somerset Health Authority in the UK has contracted a team of accredited sport and exercise scientists at the University of Gloucestershire to: ensure quality of advice from leisure providers; provide workshops for fitness professionals on current research-based knowledge to safely deal with lower risk patients; be a consultancy service; and provide bimonthly newsletters focused on information dissemination [23]. However, no formal evaluation was reported in the literature regarding the impact of these KT interventions on knowledge uptake, intention to use or actual use of the evidence in practice, or fitness trainer satisfaction with the KT strategies.

While data are available regarding KT interventions to healthcare providers [16], it is unclear whether or not these interventions would also be effective with fitness trainers. Compared to healthcare providers, fitness trainers do not have standardized levels of educational preparation, and it is unclear whether their practice is motivated by other factors (*e.g*., marketing their services, maintaining their clientele, client satisfaction). Given that one of the most common sources of information for fitness trainers is networking with peers, interventions such as communities of practice and/or using local opinion leaders may be more appealing KT approaches worth evaluating.

There are several limitations and strengths that should be considered when interpreting the findings from our systematic review. With only two studies identified, it is possible some studies were missed due to poor indexing in databases [24]. The search strategy was intended to be broad in nature and comprehensive in that we used a variety of approaches and data sources. Therefore, given the search methods and screening process using two independent reviewers, it is unlikely that many, if any, were missed. Another limitation is the descriptive nature of the available studies failing to provide highest quality evidence in support of any conclusions. Finally, we were unable to assess for publication bias due to small number of studies [25]. In addition to the rigorous and systematic methods used, another strength of our review was having an inter-professional research team with expertise in KT (DS), library sciences (RS), kinesiology (MH, DP, KA), and personal training/exercise physiology (MH, KA).

## Summary

In conclusion, there is insufficient evidence to determine how fitness trainers attain new evidence-based knowledge and incorporate this knowledge into their practice. There is no known evidence examining effective strategies to translate knowledge for this group of fitness experts. Therefore, given fitness trainers' role in advising the general public, their accessibility, and the emerging evidence-based guidelines on best practices related to the use of exercise and nutrition interventions, further research is needed to ensure that fitness trainers, working with the public, integrate new research knowledge into their fitness assessment and exercise guidance.

## Competing interests

The authors declare that they have no competing interests.

## Authors' contributions

DS and MH conceived of the study and were involved in its design, reviewing search results for eligibility, extracting data, interpretation of results, and drafting of the article. RS designed the search strategies and provided important intellectual content for the article. KA and DP participated in study design, interpretation of the results, and revised the article for important intellectual content. All authors approved of the final manuscript.
